# *PIK3CA* Mutation Analysis in Iranian Patients with Gastric Cancer

**DOI:** 10.29252/.23.1.87

**Published:** 2019-01

**Authors:** Mostafa Iranpour, Mahyar Nourian, Sana Saffari, Esmaeil Samizadeh, Mahdieh Mirghafori, Shahrokh Iravani, Soudeh Ghafouri-Fard

**Affiliations:** 1 AJA Cancer Epidemiology Research and Treatment Center (AJA-CERTC), AJA University of Medical Sciences, Tehran, Iran; 2 Department of Biology, Tehran North Branch, Islamic Azad University, Tehran, Iran; 3 Department of Pathology, AJA University of Medical Sciences, Tehran, Iran; 4 Department of Biology and Anatomical Sciences, Shahid Beheshti University of Medical Sciences, Tehran, Iran; 5 Department of Medical Genetics, Shahid Beheshti University of Medical Sciences, Tehran, Iran

**Keywords:** Mutation, Phosphatidylinositol-3 kinases, Somach neoplasms

## Abstract

**Background:**

Aberrant activation of phosphatidylinositol-3 kinases (PI3K)/AKT/mTOR (mammalian target of rapamycin) pathway is a critical event during gastric cancer progression. Selective function of AKT inhibitor AZD5363 in *PI3KCA* mutant gastric cancer necessitates the assessment of *PI3KCA* mutations in these patients.

**Methods:**

The study included 100 patients with gastric cancer who underwent surgical resection at Imam Reza Hospital, Tehran, Iran, between January 2009 and December 2016. Mutations in codon 1047 of *PIK3CA* were evaluated by tetra-primer ARMS-PCR and direct sequencing methods.

**Results:**

We detected p.H1047R and p.H1047L in eight and three samples, respectively. Also, a significant association was found between *PIK3CA* mutations and lymphatic invasion. Kaplan-Meier analysis demonstrated no significant differences in overall survival between patients with and without mutations.

**Conclusion:**

Our study detected gain-of-function mutations in exon 20 of *PI3KCA* gene in 11% of gastric cancer patients. Future studies are needed to assess the mutation rate in other regions of this gene to find eligible patients for targeted therapies.

## INTRODUCTION

Gastric cancer is regarded as one of the most frequent causes of cancer death all over the world[1]. Numerous genetic and environmental factors known to be implicated in the pathogenesis of this disease, as well as in patients’ response to treatment modalities[2]. Dysregulation of phosphatidylinositol-3 kinases (PI3K)/AKT/mTOR (mammalian target of rapamycin) signaling pathway has been recognized as the causative agent of gastric cancer pathogenesis[3]. This pathway has a crucial role in the regulation of cell growth, metabolism, vesicular trafficking, cell apoptosis, metastasis, and response to chemotherapy[4]. One of the most investigated components of this pathway is the *PIK3CA* gene encoding p110α. Activating mutations in this gene have been detected in a variety of human malignancies, leading to the constitutive expression of p110α and to the induction of downstream signaling proteins, including AKT/protein kinase B, mTOR, and ribosomal protein S6 kinase[4]. Exons 20 and 9 of this gene, which code for kinase and helical domains, respectively, are considered as mutation hotspots in cancer cells[5,6]. The advent of novel targeted therapies against PI3K pathway has further increased the significance of the assessment of *PIK3CA* gene alterations in cancers. Most notably, *PI3KCA* mutation has been identified as an essential determinant of response to AKT inhibitor AZD5363 in gastric cancer cell lines[7]. Moreover, clinical evidence supports the essential role of *PI3KCA* mutations in the determination of response to ipatasertib and AZD5363 AKT inhibitors[8]. Few studies have assessed the frequency of *PIK3CA* gene mutations in gastric cancer patients and their influence on patients’ survival[6]. However, based on the differences in etiologic factors of gastric cancer and disparities in patients’ outcome in distinct ethnicities[9], it is important to assess the rate of *PIK3CA* gene mutations in each region in association with patients’ survival. Consequently, in the present study, we evaluated the mutation status of a single hotspot codon in *PIK3CA* gene in Iranian patients with gastric cancer.

## MATERIALS AND METHODS

### Patients

The present retrospective study included 100 gastric cancer patients who underwent surgical resection at Imam Reza Hospital, Tehran, Iran, between January 2009 and December 2016. The patients were followed-up for 12 months averagely to assess their overall survival. This parameter was delineated as the time from the date of the surgery to the date of demise. Staging was carried out based on the American Joint Committee on Cancer Staging Manual (7^th^ edition)[10]. The study was approved by the ethical committee of Medical School of Islamic Republic of Iran Army (AJA University). Informed consent was obtained from all participants.

### Genomic DNA extraction

Paraffin-embedded tissue samples of surgically resected gastric cancers were assessed by a pathologist to spot cancer tissues. Genomic DNA was extracted from marked regions including tumor tissues without adjacent normal tissues using Exgene™ FFPE Tissue DNA kit (GeneAll, Korea).

### PCR amplification of codon 1047 of *PI3KCA* gene

Primers for tetra-primer ARMS-PCR were designed by PRIMER1 online tool[11]. Table 1 shows the nucleotide sequences of primers used in the current study. The PCR reaction was performed in a 25-µl total volume containing 100 ng of genomic DNA, 12.5 µl of Taq DNA Pol 2× Master Mix Red (Ampliqon, Denmark), and 0.5 µl of inner and outer primers. Then 10% of the samples were sequenced by using the ABI 3730xl DNA analyzer (Macrogen, Korea) in order to confirm the results of tetra-primer ARMS-PCR.

**Table 1:** The nucleotide sequences of primers used in this study

Primers	Primer sequence	Product length (bp)
Inner	F: 5’-ATTTCATGAAACAAATGAATGATGCACA-3’ (A-allele)	149
	R1: 5’-CCATTTTTGTTGTCCAGCCACCATGAC-3’ (G-allele)	198
	R2: 5’-CCATTTTTGTTGTCCAGCCACCATGAA-3’ (T-allele)	198
Outer	F: 5’-CTATTCGACAGCATGCCAATCTCTTCAT-3’	292
	R: 5’-TTAACAGTGCAGTGTGGAATCCAGAGTG-3’	

### Statistical analysis

The results were statistically analyzed by SPSS software (version 16, Chicago, USA) using *t*-test and chi-square test (χ^2^) for comparing means and proportional differences among the clinical features. Survival time was assessed by the Kaplan-Meier method and compared using the log-rank test.

## RESULTS

### *PIK3CA* codon 1047 mutations in gastric cancer patients

**Fig. 1:**
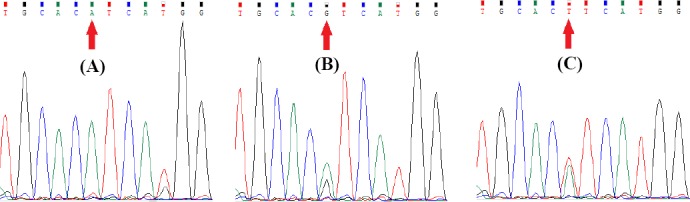
The results of Sanger sequencing for confirmation of tetra-primer ARMS-PCR. The normal sequence (A), c.3140A>G (B), and c. 3140A>T (C) mutations.

Among the 100 patients who had undergone gastric resection, 11 (11%) were recognized by tetra-primer ARMS-PCR and also direct sequencing to harbor *PIK3CA* codon 1047 mutations (Fig. 1). Eight patients were positive for c.3140A>G (p.H1047R) and three were positive for c.3140A>T (p.H1047L) mutations.

### *PIK3CA* codon 1047 mutations and patients’ clinical and demographic data

Table 2 shows the details of statistical analysis of the associations between mutation status and patients’ demographic and clinical data. No significant association was found between mutation status and patients’ age or gender. However, a significant correlation was detected between the presence of mutation and lymph node involvement (*p* = 0.036).

**Table 2:** Associations between mutation status and patients’ demographic and clinical data

Characteristics	Mutation (%) (n = 11)	Wild-type (%) (n = 89)	*p* value
Age (years)	61.55 ± 11.50	62.87 ± 9.92	0.683
Gender			
Female	5 (5)	33 (33)	0.589
Male	6 (6)	56 (56)	
Lymph node involvement			
Positive	8 (8)	37 (37)	0.036
Negative	2 (2)	39 (39)	
Missing	1 (1)	13 (13)	
Familial history of cancer			
Positive	1 (1)	7 (7)	0.833
Negative	4 (4)	36 (36)	
Missing	6 (6)	46 (46)	

### *PIK3CA* codon 1047 mutations and patients’ survival

**Fig. 2:**
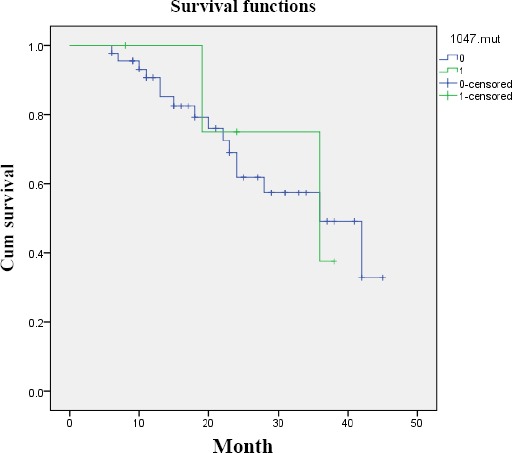
Kaplan-Meier curves showing the relationship between codon 1047 mutational status and the overall survival of gastric cancer patients.

We evaluated the effect of *PIK3CA* codon 1047 mutations on patients’ survival after the surgical resection of gastric cancer. The median follow-up time for patients was 20 months. Kaplan–Meier analysis showed no significant differences in overall survival (log rank *p* = 0.55) between the patients with and without codon 1047 mutations (Fig. 2).

## DISCUSSION

In the present study, we demonstrated the presence of activating mutations in a single codon of *PIK3CA* gene in a significant number of Iranian patients with gastric cancer. Previous studies have detected mutations in other regions of this gene in gastric cancer samples[5,6]. Our detected mutation rate in this single codon was comparable with the total rate of detected mutations in two *PIK3CA* exons in a study conducted in Japanese patients[6], implying the presence of higher mutation rate in entire gene in Iranian patients. Harada *et al*.[6] have analyzed exons 9 and 20 of this gene in Japanese patients with gastric cancer. They reported a total mutation rate of 12% with p.H1047R and p.H1047L being seen in 4% and 0% of patients. Sukawa *et al*.[12] have detected *PIK3CA* mutations in 8.7% of Japanese gastric cancer patients in their assessment of exons 1, 9, and 20 and reported p.H1047R and p.H1047L mutations in 2.6% and 0% of patients, respectively. Besides, Lee *et al*.[13] have reported a total rate of 7.7% for exons 9 and 20 mutations in Korean gastric cancer patients. Meanwhile, Barbi *et al*.[14] have reported a total mutation rate of 16% in mutation analysis of exons 9 and 20 in Italian gastric cancer patients with p.H1047R, being the most prevalent mutation. Our data are in accordance with the reported data in gastric cancer patients regarding the common occurrence of p.H1047R mutation. However, p.H1047L mutation has not been previously reported in gastric cancer.

Evaluation of biological and biochemical characteristics of p.H1047L, p.H1047Y, and p.H1047R mutations in the kinase domain of *PIK3CA* gene have shown that all of these mutations pack against the hinge region of the activation loop and trigger rapamycin-sensitive oncogenic transformation of chicken embryo fibroblasts through the induction of AKT and mTOR signaling[15]. Consequently, no functional difference is predicted to occur in patients carrying any of these mentioned mutations.

Previous studies have reported no associations between *PIK3CA* gene mutations and gastric patients’ clinicopathologic features[6,12,14]. Consistent with these investigations, we observed no significant association between *PIK3CA* gene mutations and patients’ survival, which might be due to the occurrence of these mutations in the early stages of carcinogenesis that are shared between all patients. Therefore, other factors might override this event and change the disease course. In contrast, we found a significant correlation between the occurrence of codon 1047 mutations and lymph node involvement. As a result, we suggest the evaluation of other hotspots in *PIK3CA* gene to assess the clinical significance of such finding.

Assessment of somatic *PIK3CA* mutations has been suggested as an effective biomarker for recruitment of patients in clinical trials of AKT inhibitors[8]. The high incidence of mutation in one codon of *PIK3CA* gene in our assessed population of gastric cancer patients warrants the evaluation of total coding region of this gene in Iranian patients to confirm the contribution of these mutations in gastric cancer and to develop personalized therapeutic regimens for patients.
